# Systemic metabolite profiling reveals sexual dimorphism of AIBP control of metabolism in mice

**DOI:** 10.1371/journal.pone.0248964

**Published:** 2021-04-01

**Authors:** Jun-dae Kim, Lingping Zhu, Quan Sun, Longhou Fang

**Affiliations:** 1 Center for Cardiovascular Regeneration, Houston Methodist Research Institute, Houston, TX, United States of America; 2 Department of Geriatric Medicine, Xiangya Hospital, Central South University, Changsha, Hunan, P.R. China; 3 Department of Cardiovascular Sciences, Houston Methodist Research Institute, Houston, TX, United States of America; 4 Weill Cornell Medical College, New York, NY, United States of America; Laurentian University, CANADA

## Abstract

Emerging studies indicate that APOA-I binding protein (AIBP) is a secreted protein and functions extracellularly to promote cellular cholesterol efflux, thereby disrupting lipid rafts on the plasma membrane. AIBP is also present in the mitochondria and acts as an epimerase, facilitating the repair of dysfunctional hydrated NAD(P)H, known as NAD(P)H(X). Importantly, AIBP deficiency contributes to lethal neurometabolic disorder, reminiscent of the Leigh syndrome in humans. Whereas cyclic NADPHX production is proposed to be the underlying cause, we hypothesize that an unbiased metabolic profiling may: 1) reveal new clues for the lethality, e.g., changes of mitochondrial metabolites., and 2) identify metabolites associated with new AIBP functions. To this end, we performed unbiased and profound metabolic studies of plasma obtained from adult AIBP knockout mice and control littermates of both genders. Our systemic metabolite profiling, encompassing 9 super pathways, identified a total of 640 compounds. Our studies demonstrate a surprising sexual dimorphism of metabolites affected by AIBP deletion, with more statistically significant changes in the AIBP knockout female vs male when compared with the corresponding controls. AIBP knockout trends to reduce cholesterol but increase the bile acid precursor 7-HOCA in female but not male. Complex lipids, phospholipids, sphingomyelin and plasmalogens were reduced, while monoacylglycerol, fatty acids and the lipid soluble vitamins E and carotene diol were elevated in AIBP knockout female but not male. NAD metabolites were not significantly different in AIBP knockout vs control mice but differed for male vs female mice. Metabolites associated with glycolysis and the Krebs cycle were unchanged by AIBP knockout. Importantly, polyamine spermidine, critical for many cellular functions including cerebral cortex synapses, was reduced in male but not female AIBP knockout. This is the first report of a systemic metabolite profile of plasma samples from AIBP knockout mice, and provides a metabolic basis for future studies of AIBP regulation of cellular metabolism and the pathophysiological presentation of AIBP deficiency in patients.

## Introduction

Cellular metabolites serve as the engine to drive distinct cell functions. Our and other publications have shown that AIBP functions as a critical component of lipid metabolism [1–17]. AIBP is a secreted protein that interacts with APOA1/HDL and promotes HDL binding to the endothelial cells (ECs) while accelerating the HDL-EC dissociation [18], which promotes free cholesterol (shortened as cholesterol hereafter) efflux from ECs and macrophages, resulting in cholesterol-rich lipid raft/caveolae disruption [2, 4, 9, 13, 16]. Lipid rafts serve as the critical platform for the clustering and proper function of many plasma membrane receptors, including angiogenic VEGFR2 signaling and TLR4-mediated inflammatory response. Both signaling are repressed by AIBP gain-of-function [2, 4, 5, 9, 12, 13, 17]. Raising AIBP levels ameliorates diet-induced metabolic syndrome and reduces atherosclerotic burden in *Ldlr*^*-/-*^ mice that were fed western diet [9, 14]. We have recently shown that AIBP-mediated cholesterol efflux activates sterol regulatory element binding protein 2 (SREBP2), which in turn transcriptionally upregulates NOTCH1, thereby connecting hematopoiesis and hypercholesterolemia [6]. Low et al. suggest an alternative mechanism for AIBP regulation of lipid rafts: they reported that AIBP per se, but not cholesterol efflux, modulates lipid raft in macrophages via the cellular cytoskeleton; in this scenario, AIBP binding of PI3P regulates Rho family GTPase CDC42 activation, thereby resulting in cytoskeleton rearrangement and lipid raft disassembly [7].

**Fig 1:**
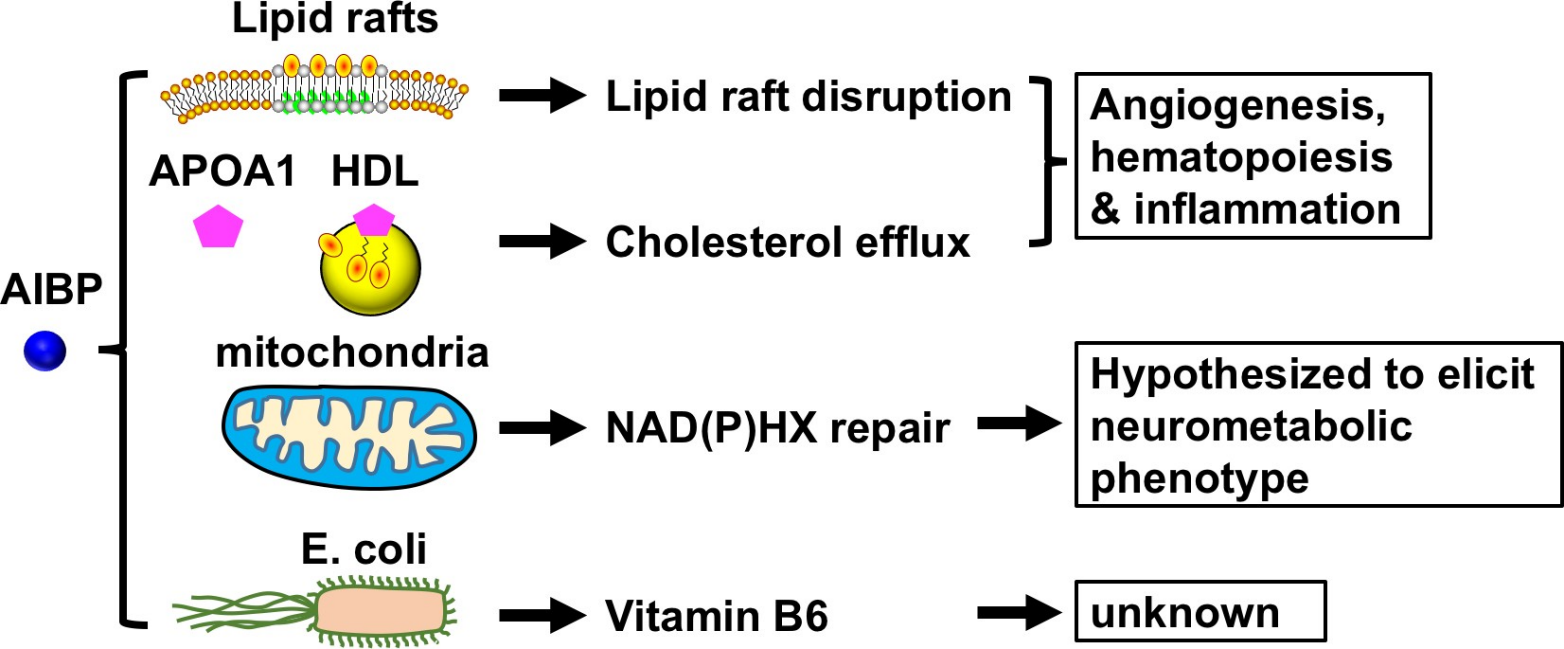
Summary of the current understanding of the AIBP role in metabolism and physiology. AIBP can disrupt lipid rafts via cholesterol efflux dependent or independent mechanism, which contributes to regulation of angiogenesis, hematopoiesis, and inflammation. AIBP localization in the mitochondria is involved in NAD(P)HX repair. The AIBP paralog of E. coli may regulate Vitamin B6 biosynthesis.

Another function of AIBP relates to its intracellular localization in the mitochondria and its role as a NAD(P)HX epimerase [19]. Cellular NAD(P)H can be hydrated to R or S configuration of NAD(P)HX by heat or GAPDH; only the S but not R epimer can be de-hydrolyzed and reverted to the metabolically active NAD(P)H [20]. Thus, AIBP is hypothetically critical for the maintenance of the homeostasis of NAD(P)H. Several human genetic studies ascribed a lethal neurometabolic disorder of AIBP deficiency to toxic cyclic NAD(P)HX production [21–24]. The clinical presentation of AIBP deficiency is similar to that of Leigh syndrome, a disease caused by the dysfunction of mitochondrial complex I [25]. Consistent with this, AIBP knockout reduces OXPHOS complex I levels in mice [26]. In addition, AIBP has been reported to function in vitamin B6 metabolism, a function that is independent of the epimerase activity [27]. The current understanding of AIBP function is summarized in Fig 1.

Given these distinct roles of AIBP in extracellular, cytosolic and mitochondrial functions [4, 27, 28], we sought to systemically characterize the metabolites of plasma samples obtained from a cohort of 40 mice, which included 20 adult AIBP knockout mice and 20 control littermates of both genders. Our untargeted profiling of metabolites contributes to the understanding of AIBP function in health and disease.

## Results

### Profound gender effects observed in the AIBP knockout mice

**Fig 2:**
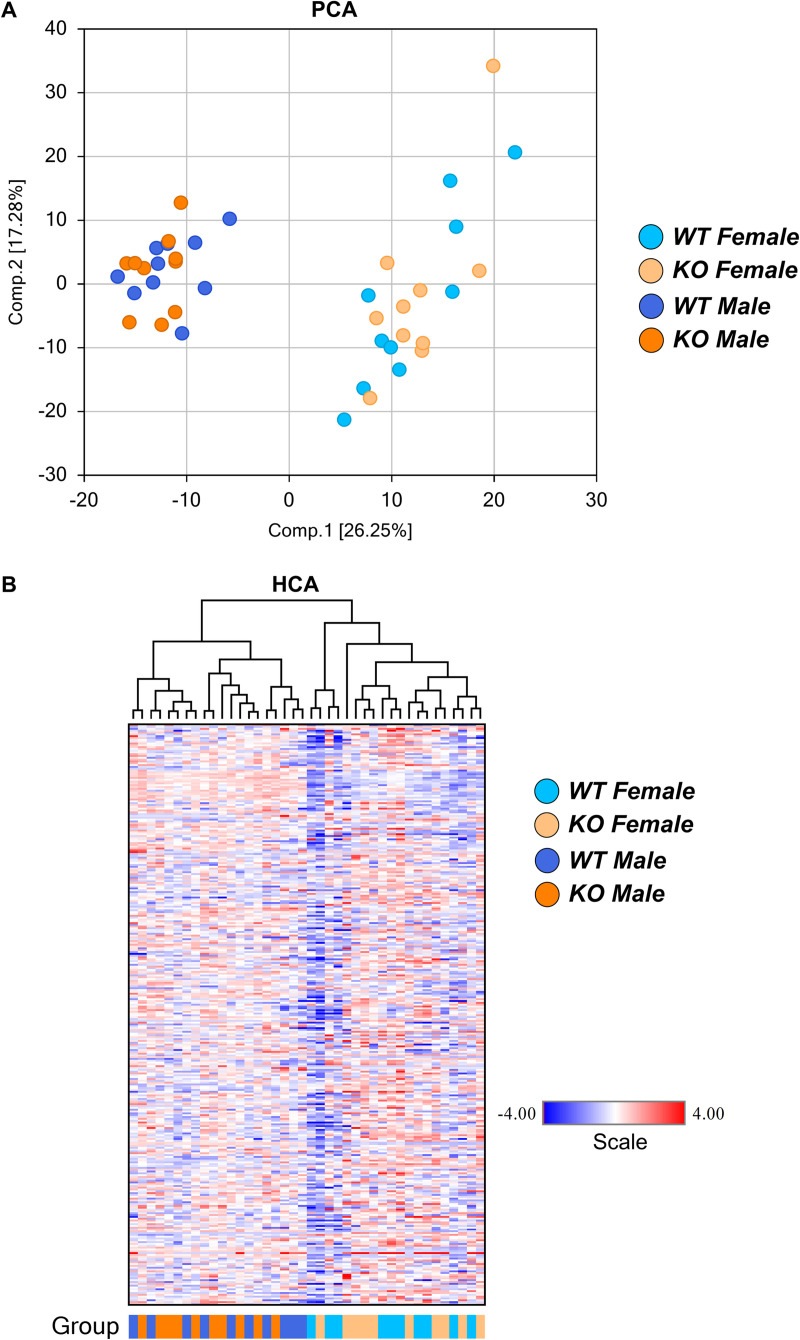
Bioinformatics analysis of metabolites. Fifty to 100 µl of plasma were drawn from mice and subjected to untargeted metabolite analysis by tandem LC/MS. PCA (**A**) and HCA (**B**) analysis of AIBP-regulated metabolites in the plasma of AIBP knockout (KO) and control littermate mice.

AIBP regulates metabolism in multiple tissues, such as blood vessels, leukocytes, liver, lung, and brain [2, 4, 9, 15, 17, 21]. To gain a holistic view, we profiled metabolites in murine plasma of control and AIBP knockout adult mice. Mice were fasted for 6 hours and plasma collected via jugular vein from the live animals. Tandem liquid chromatography-mass spectrometry (LC-MS/MS) was performed with internal standards included. A total of 640 known metabolites were identified using untargeted primary metabolite profiling. Only a small number of metabolites were changed in the AIBP null mice compared with sex-matched controls. Principle component analysis (PCA) and hierarchical cluster analysis (HCA) of the metabolite profile showed that AIBP knockouts and control littermates were clustered more by gender than by AIBP expression status (Fig 2A and 2B). More statistically significant differences (p<0.05) were detected in female than in male knockout mice vs sex-matched control mice (36 vs 8, Table 1). Our data show that AIBP deficiency elicits a marginal change of biochemical content in the plasma, with more changes observed in the female animals.

**Table 1:** Summary of changes of AIBP-associated metabolites in AIBP knockout (KO) and Control Littermate (CT) mice.

Statistical Comparisons				
ANOVA analysis	KO: CT (female)	KO: CT (male)	female:male (CT)	female:male (KO)
**Total biochemical** **p≤ 0.05**	36	8	314	319
**Biochemicals (↑↓)**	16 | 20	5 | 3	143 | 171	143 | 176
**Total biochemical** **0.05<p<0.10**	24	31	41	46
**Biochemicals (↑↓)**	10 | 14	17 | 14	21 | 20	23 | 23

↑ indicates increased metabolites, and ↓ denotes reduced metabolites.

### AIBP deletion affects plasma sterol concentration in female but not male mice

**Fig 3:**
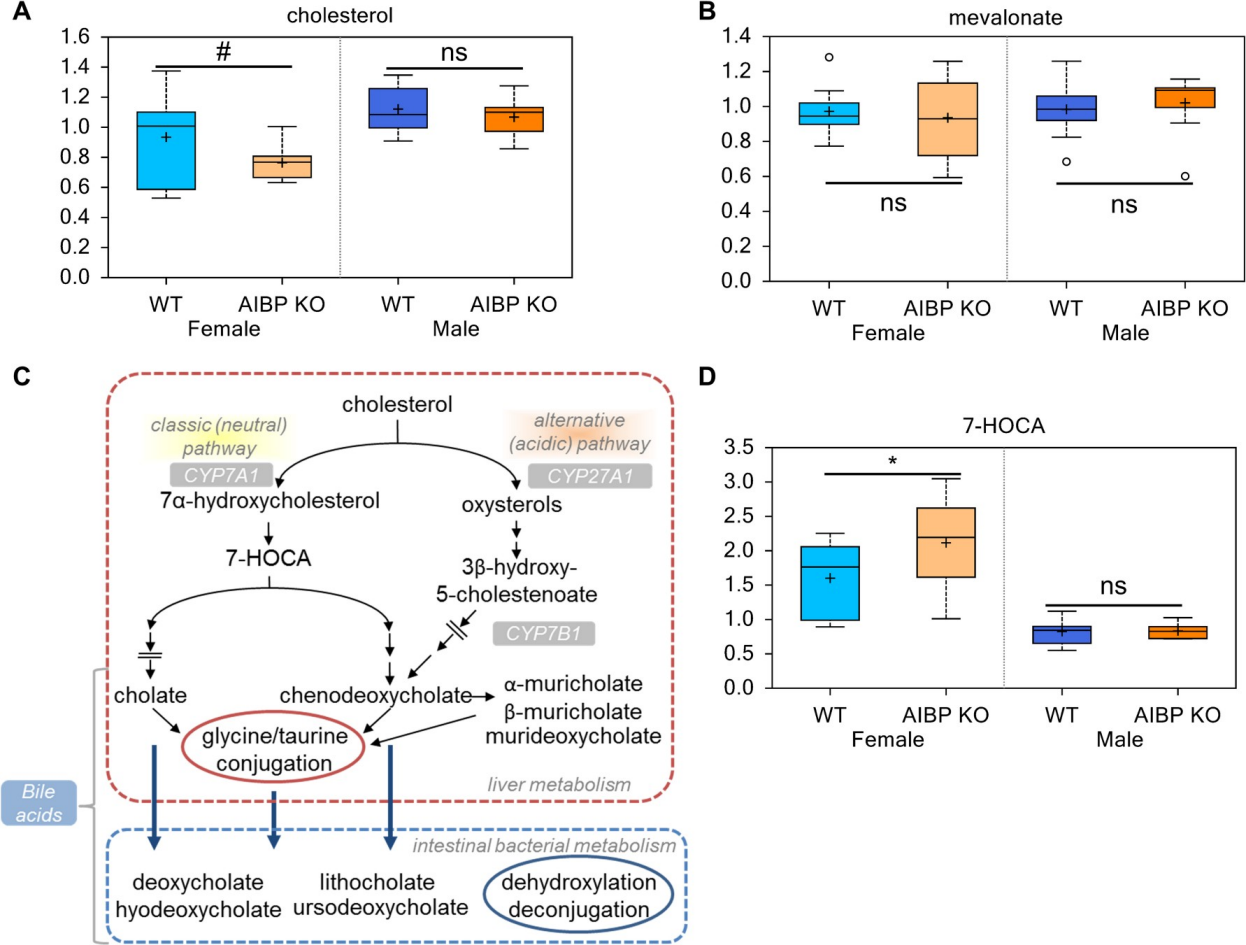
AIBP regulation of cholesterol and bile acid metabolism. Cholesterol (**A**) and its precursor mevalonate (**B**) content detected in the control and AIBP knockout (KO) mice. **C**. The two biochemical pathways for the biosynthesis of primary and secondary bile acids. Plasma content of 7-HOCA (**D**). *, p<0.05. #: 0.05<p<0.1. ns: not significant (p>0.1).

We observed that plasma cholesterol levels trended lower in female AIBP knockout vs littermate control group (0.82 fold; p = 0.08) (Fig 3A), while cholesterol levels were not affected in male mice. Mevalonate, the precursor for cholesterol biosynthesis, was not changed by AIBP knockout (ratio of 0.96 in females and 1.04 fold in males) (Fig 3B). Cholesterol can be converted to primary and secondary bile acids; biosynthesis of the former is initiated in the liver by CYP7A1 and CYP27A1 (Fig 3C), while the latter is synthesized by the gut microbiota [29]. AIBP knockout increased 7-alpha-hydroxy-3-oxo-4-cholestenoate (7-HOCA), a precursor of bile acid biosynthesis in female mice (1.32 fold; p<0.05) (Fig 3D). AIBP knockout did not affect the levels of 7-HOCA in male mice (Fig 3D). Consistent with this, both primary (e.g., taurocholate and taurochenodeoxycholate) and secondary (e.g., tauroursodeoxycholate and 7-ketodeoxycholate) bile acids trended higher in the AIBP knockout female group, although this did not reach statistical significance (S1 Table). Thus, cholesterol metabolism is altered in female but not male AIBP knockout mice.

### AIBP knockout modulates lipid content in female but not male mice

**Fig 4:**
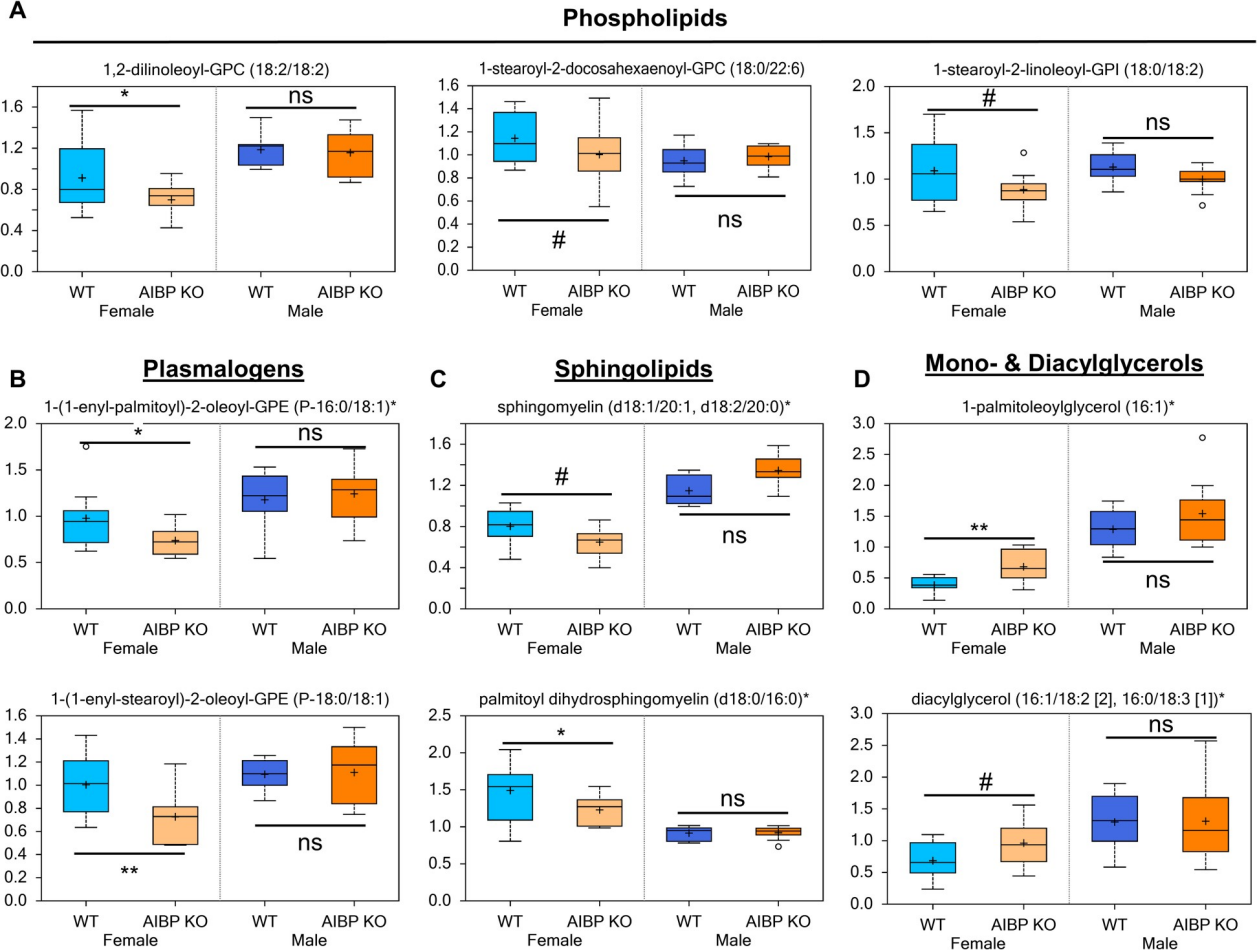
Effects of AIBP deficiency on complex lipid content. Plasma content of phospholipids (A), plasmalogens (B), sphingolipids (C), and mono- and diacylglycerols (D). **,p<0.01; *, p<0.05. #: 0.05<p<0.1. ns: not significant (p>0.1).

Several phospholipid species were lower or trended lower in AIBP knockout female mice. These included phosphatidylcholines, e.g., 1,2-dilinoleoyl-GPC (18:2/18:2) (0.77 fold; p<0.05), 1-stearoyl-2-docosahexaenoyl-GPC (18:0/22:6) (0.88 fold; p = 0.087), and phosphatidylinositols, e.g., 1-stearoyl-2-linoleoyl-GPI (18:0/18:2) (0.81 fold; p = 0.095) (Fig 4A, S1 Table). In addition, several plasmalogen species, e.g., 1-(1-enyl-palmitoyl)-2-oleoyl-GPE (P-16:0/18:1) (0.75 fold; p<0.05) and 1-(1-enyl-stearoyl)-2-oleoyl-GPE (P-18:0/18:1) (0.72 fold; p<0.01) were lower in the plasma from AIBP null female relative to control mice (Fig 4B).

Sphingolipids, which are enriched in lipid rafts, were also lower in AIBP null female but not male, e.g., palmitoyl dihydrosphingomyelin (d18:0/16:0) (0.83 fold; p<0.05) and sphingomyelin (d18:1/20:1, d18:2/20:0) (0.87 fold; p = 0.055) (Fig 4C). In contrast to membrane lipids, monoacylglycerols [MAGs, e.g., 1-palmitoleoylglycerol (16:1)] (1.77 fold; p<0.01) were elevated and diacylglycerols [DAGs, e.g., diacylglycerol (16:1/18:2 [2], 16:0/18:3 [1])] (1.41 fold; p = 0.069) trended higher in the AIBP knockout female vs control mice (Fig 4D). These changes may be indicative of the increased hydrolysis of triacylglycerols (TAGs) to DAGs and MAGs.

**Fig 5:**
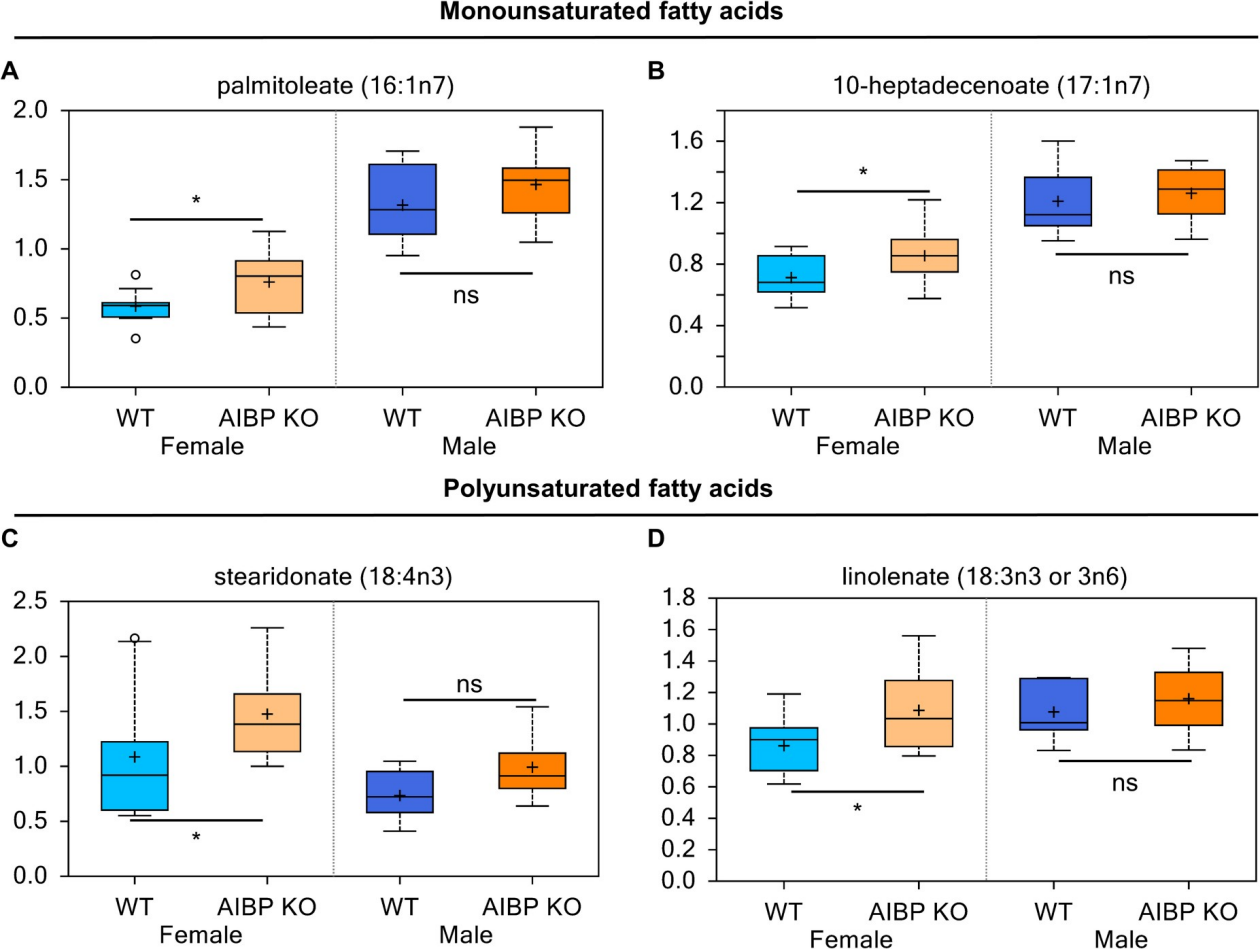
AIBP effect on mono- and polyunsaturated fatty acid metabolism. Changes of plasma content of monounsaturated fatty acids (A & B) and polyunsaturated fatty acids (C&D). *, p<0.05. ns: not significant (p>0.1).

In addition, we detected higher levels of mono- and polyunsaturated fatty acids, e.g., palmitoleate (16:1n7) (1.30 fold; p<0.05), 10-heptadecenoate (17:1n7) (1.20 fold; p<0.05), stearidonate (18:4n3) (1.36 fold; p<0.05), and linolenate [α or γ; (18:3n3 or 6)] (1.26 fold; p<0.05) in the AIBP knockout female compared to control mice (Fig 5A–5D). Our results suggest that AIBP deletion modulates lipid metabolism more in female than male mice.

### Alterations in lipid-soluble vitamins

**Fig 6:**
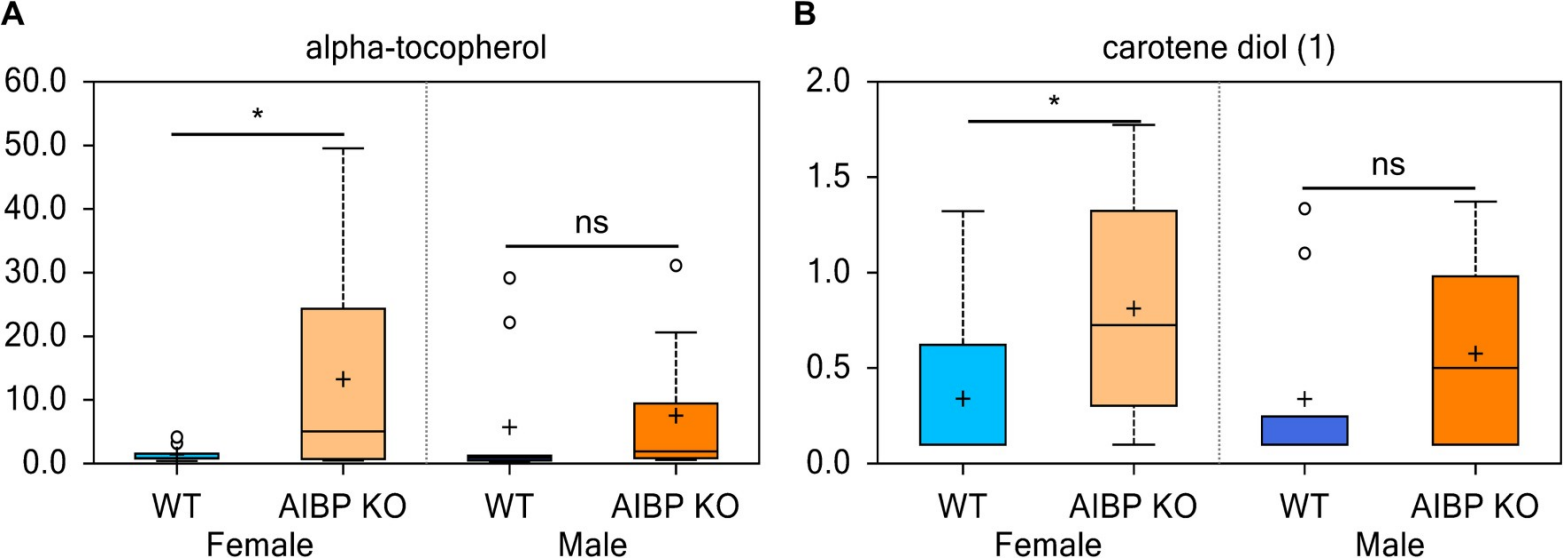
Effects of AIBP on lipid soluble vitamin metabolism. Measurements of plasma content of lipid soluble vitamin E (**A**) and vitamin A precursor (**B**). *, p<0.05 ns: not significant (p>0.1).

In humans, plasma HDL and LDL are the major carriers for α-tocopherol, the major form of vitamin E that can function as an antioxidant to actively prevent lipid peroxidation. In mice, HDL is the primary vehicle for lipophilic vitamins in females while LDL transports the majority of these vitamin in males [30]. We found that the mean content of α-tocopherol was increased over 9-fold in female AIBP knockout vs control (Fig 6A) (p<0.05). Although retinol levels (vitamin A) showed only slight changes in the current study, the levels of provitamin A carotene diol (1) were substantially increased (2.39-fold, p<0.05) in the female AIBP knockout vs control (Fig 6B). The carotenoids are transported in plasma primarily by LDL and to a lesser extent by HDL [31]. All together, these differences in lipid-soluble vitamins suggest alterations in HDL levels, composition, and/or turnover rates in female mice lacking AIBP.

### Nicotinamide levels trends lower in male but not female AIBP knockout mice

Our goal for this study was to capture the change of global metabolic spectrum of AIBP deletion in live animals, we thus collected blood via the jugular vein. This blood collection method introduced some hemolysis. As such, we detected NAD+, NADH, and NADPH, typically not present in plasma samples. We also identified the metabolite 2,3-diphosphoglycerate that is generated in the Rapoport Leubering cycle during erythrocyte glycolysis [32]. Although hemolysis introduces metabolites of erythrocytes, this did not affect our comparisons of AIBP knockout to control samples because the erythrocyte derived 2,3-diphosphoglycerate was present at similar levels in AIBP knockouts vs control animals (1.17 fold in female group and 0.52 fold in male group, p>0.1, S1 Table).

**Fig 7:**
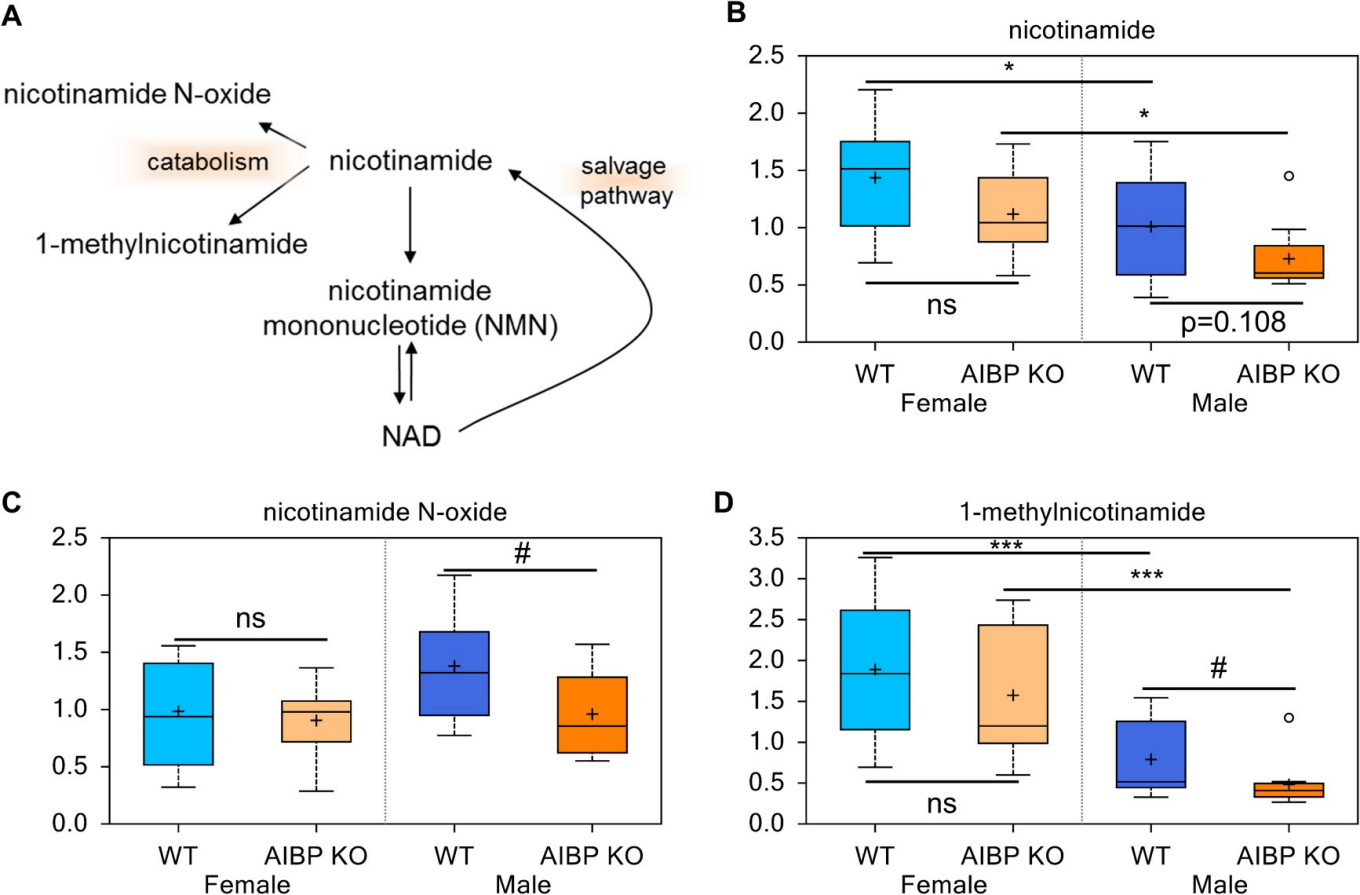
AIBP modulation of NAD metabolism. **A.** NAD biosynthesis pathways. Measurements of plasma content of nicotinamide (**B**) nicotinamide N-oxide (**C**), and 1-methylnicotinamide (**D**). #: 0.05<p<0.1; ns: not significant (p>0.1). ***:p<0.001.

AIBP can catalyze the epimerization of NAD(P)HX from the R to S epimer, which subsequently can be converted by dehydratase to metabolically active NAD(P)H [20]. The involvement of AIBP in NAD(P)H metabolism provoked us to assess their precursors and immediate metabolites (Fig 7A). Nicotinamide, the precursor for NAD+ biosynthesis (0.72 fold; p = 0.108), nicotinamide N-oxide (0.70 fold; p = 0.064) and 1-methylnicotinamide (0.61 fold; p = 0.066) trended lower in AIBP knockout vs control males (Fig 7B–7D). Nicotinamide levels were significantly higher in female than that in male mice of both genotypes (1.42 fold; p<0.05 for control and 1.54 fold; p<0.05 for the knockout) (Fig 7B), and this was also the case for 1-methylnicotinamide (Fig 7D). Given the essential role of NAD/NADH in glycolysis and the Krebs cycle, we compared metabolites associated with glycolysis: glucose, 2, 3-diphosphoglycerate, 3-phosphoglycerate, pyruvate and lactate (S1A-S1E Fig in S1 File), and the Krebs cycle: citrate, aconitate (cis or trans), isocitrate, alpha-ketoglutarate, succinate, fumarate, malate, and tricarballylate (S2A–S2H Fig in S1 File and S1 Table). Of these, only 3-phospholycerate (S1D Fig in S1 File) was significantly lower in AIBP KO female; none of the other metabolites was altered in the AIBP knockout mice. Thus, AIBP deletion appears to have minimal effects on glycolysis or the Krebs cycle when analyzed at the systemic level.

### Changes in tryptophan metabolism

**Fig 8:**
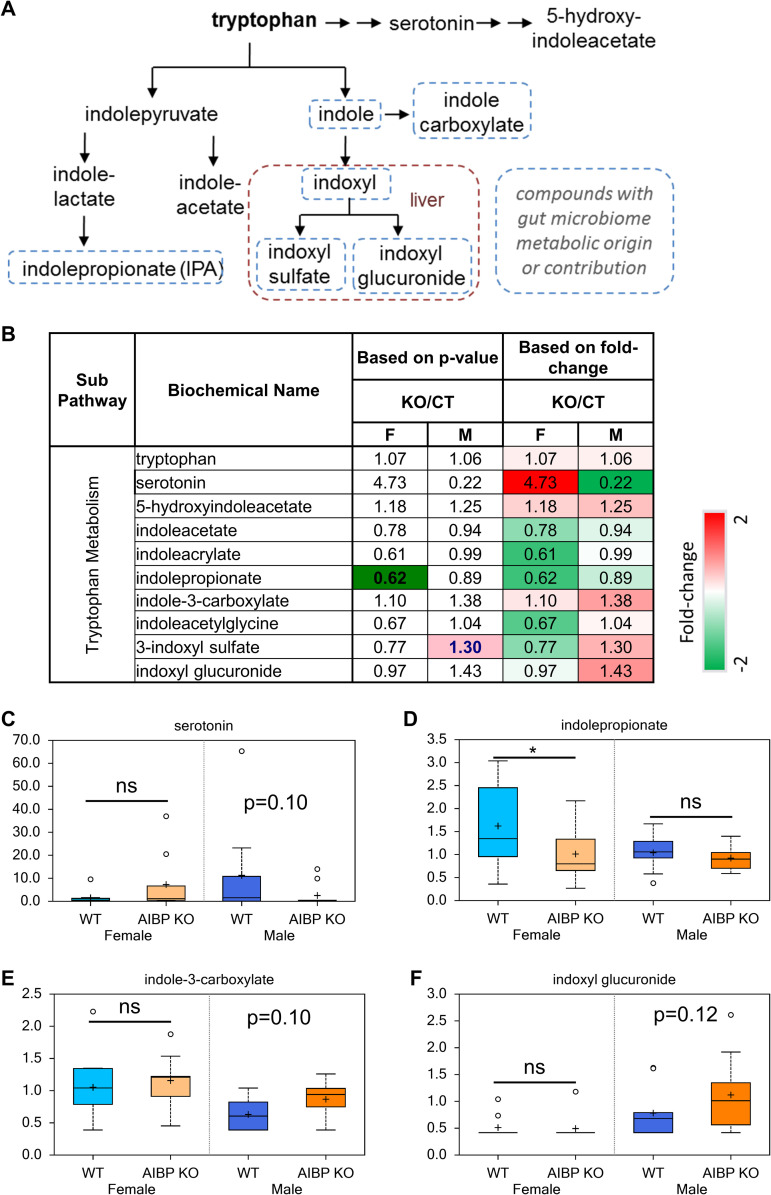
AIBP and tryptophan metabolism. **A**. Tryptophan biosynthesis pathways. **B**. Fold change of tryptophan metabolites in control and AIBP knockout mice. Dark green indicates reduction with statistical significance p<0.05, light green show reduction with statistical significance 0.05<p<0.1. Dark red denotes increase with statistical significance p<0.05, light red highlights increase with statistical significance 0.05<p<0.1. *: p<0.05; ns: not significant (p>0.1).

Genetic deletion of AIBP altered circulating serotonin levels in a gender-specific manner: while the mean serotonin levels were 4.7-fold higher in AIBP knockout female vs control mice, the wide range of individual values prevented this from being statistically significant. The content in male knockout were lower, but was not significantly different from that in control mice (Fig 8A–8C; p>0.1). The majority of serotonin in the body is produced in the gut by enterochromaffin cells [33]. Interestingly, gut microbiota can actively modulate biosynthesis of this neurotransmitter by the host cells [34]. Indolepropionate (IPA) was significantly reduced in the female AIBP knockout vs control mice (0.62; p<0.05) (Fig 8D), whereas indole-3-carboxylate (1.38 fold; p>0.1), indoxyl sulfate (1.30 fold; p = 0.09) and indoxyl glucuronide (1.43 fold; p>0.1) were not significantly different (Fig 8E and 8F). Further studies are needed to determine if AIBP deletion alters gut microbiota composition/activity that differentially impacts male and female animals.

### Polyamine spermidine levels is reduced in AIBP knockout male mice

**Fig 9:**
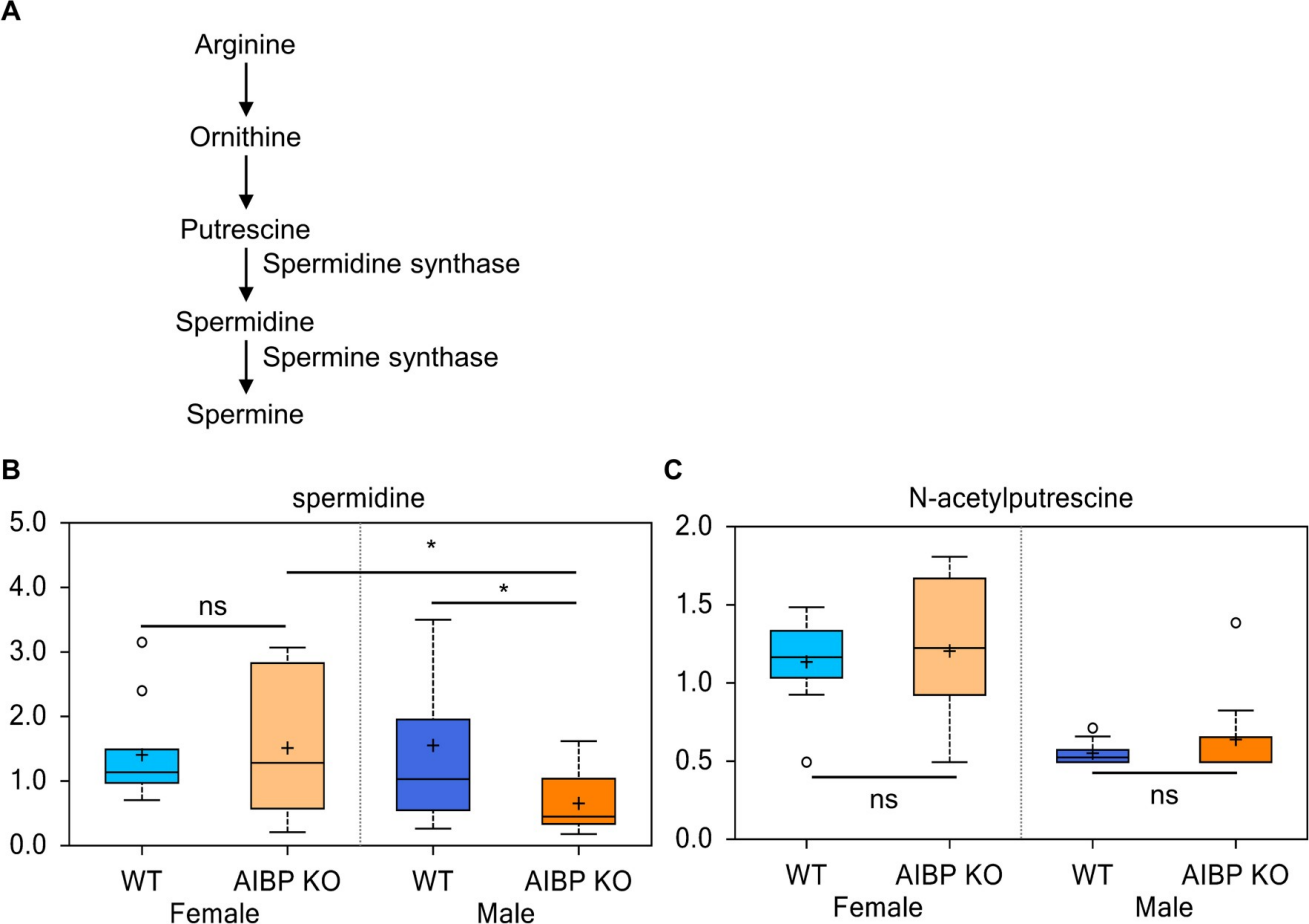
AIBP modulation of polyamine spermidine metabolism. **A**. Spermidine and spermine biosynthesis pathway. **B**. Plasma content of spermidine and its precursor derivative N-acetyl putrescine in control and AIBP knockout mice. *: p<0.05; ns: not significant (p>0.1).

We found that the polyamine spermidine is reduced in AIBP knockout male (0.42 fold, p<0.05) but not female (Fig 9A and 9B). Spermidine content is 2.31-fold higher in AIBP knockout female than that in AIBP knockout male (2.31-fold, p<0.05. Fig 9B). The precursor for spermidine biosynthesis, N-acetylputrescine, was not changed (ratios of 1.06 in males and 1.16 in females vs their respective controls) (Fig 9C). These data indicate an unexpected connection of AIBP with polyamine metabolism in male mice.

## Discussion

Given the profound neurometabolic disorder observed in patients with AIBP loss-of-function, the goal of this study is to define the major changes of AIBP-associated metabolites in an unbiased and systemic fashion, such as the mitochondrial metabolites, which likely contribute to the understanding of this lethal disease. Whereas this primary goal appears unmet, we have validated previous reports and uncovered new findings. Our untargeted metabolic study of the plasma isolated from AIBP null mice and littermate controls underscores the importance of AIBP in cholesterol metabolism and the associated derivative, e.g., bile acid. Intriguingly, some of those changes occur at the interface where cholesterol metabolism involves microbiota. Considering the conserved role of AIBP across evolution, it implicates a potential AIBP function in microbiota-host communication. Global AIBP deficiency also affects the abundance of complex lipid and lipophilic vitamin, all of which may in turn regulate lipid rafts and/or cellular signaling circuits. In contrast to the striking phenotypic penetrance of AIBP mutation in human, the overall metabolite change resulting from AIBP knockout is not substantial in these naive mice. AIBP has been implicated to bind NAD [35] and, as suggested in a recent study, control the abundance OXPHOS complex I in mitochondria [26]. While the median levels of NAD are reduced in the AIBP knockout mice (Fig 7), this change did not reach a statistical significance. A stress challenge may reveal the difference, such as in glaucoma [36], where AIBP deficiency elicits profound defects in the mitochondrial structure and function.

Our characterization of AIBP-associated metabolites was performed in the plasma. This analysis was designed to reveal systemic changes by AIBP knockout. However, for a particular organ or a specific cell type, the content of AIBP-regulated metabolites may not necessarily parallel the changes in plasma. Future studies are warranted to identify the organ-specific metabolic change, such as the brain, which may help identify the etiological mechanism in patients lacking functional AIBP. However, due to the absence of proper animal models to recapitulate the human AIBP phenotype, this makes potential translation of any of these findings challenging. A robust cause-effect relationship needs a rescue experiment at both the cellular and organism level, i.e., correction of neurometabolic disorder and the associated clinical manifestation. In this regard, if AIBP mutation results in a loss-of-function but not dominant negative effect, restored expression of wild type AIBP would be a valid option.

So far, there is a paucity of studies on AIBP [37]. We are the first to report a gender effect of AIBP regulation of metabolism. Sex hormone, such as estrogen, likely contributes to the sex dimorphism. In support of this, PCA analysis shows two distinct clusters of male and female associated metabolites (Fig 2). While several patients with AIBP deficiency have been identified, these cases appeared equally in both genders [21, 22]. However, there are considerable differences in the age of onset, disease severity, and clinical presentation of AIBP deficiency. The differential and residual AIBP expression may mask the gender effect, or alternatively, the gender effect is separated from the neurometabolic defects. To determine whether there is a similar gender preference in patients with AIBP mutation, it requires systemic characterization of biochemical and clinical components.

Prior studies have implicated a moonlighting function of bacterial AIBP paralog in B6 vitamin biosynthesis [27]. Notably, in plants, AIBP paralog is expressed as a fusion protein with the B_6_ salvage enzyme. Mammals, however, lack the capacity synthesizing B6 vitamin [38]; symbiosis between microbiota and the mammal host might contribute to the loss of AIBP regulation B6 vitamin.

## Conclusions

Our comprehensive metabolite analysis in mice detects sex dimorphism of AIBP effects on systemic metabolism. Our studies validated the reported effects of AIBP on lipid and NAD metabolism. Furthermore, our data identified new metabolites associated with AIBP deletion in a sex-specific manner, such as 7-HOCA and spermidine. Future studies on AIBP-regulated metabolites may contribute to the integration of the pathological manifestations of AIBP mutation and AIBP-regulated cellular metabolism.

## Material and methods

### Mice

2–3 months old 20 *Apoa1bp*^*-/-*^ and 20 littermate control (*C57BL/6*) mice (20 male and 20 female) were generated from *Apoa1bp*^*+/-*^ incross in our animal facility dedicated to mouse study. Veterinary care is provided on a 24 hours basis, including weekends and holidays, by a staff of veterinarians with specialties in laboratory animal medicine and anesthesiology, and licensed animal health technicians. All animal experiments were approved by the Institutional Animal Care and Use Committees (IACUC) at the Houston Methodist Research Institute.

Animals were fasted for 6 hours before drawing blood. Mice were subjected to isoflurane-mediated anesthesia in preparation for blood collection from the jugular vein. Mice were returned to their cages and fed food and water ad libitum afterwards.

### Sample processing and metabolite identification

Sample processing and mass spectrometry identification of metabolites were conducted at Metabolon Inc.

### Hierarchical clustering and Principal Components Analysis (PCA)

The bioinformatics analysis, including hierarchical clustering and PCA analysis, were carried out the bioinformatics team at Metabolon using the LAN backbone and Oracle-based analysis.

### Statistical analysis

To avoid any potential bias, statistical analysis was conducted by statistician at Metabolon Inc using ArrayStudio with log transformed data.

## Supporting information

S1 Table (XLSX)

S1 File (PDF)

## References

[1] Chen H, Yin K. AIBP, inflammation, and atherosclerosis. J Lipid Res. 2018;59(7):1081–3. Epub 2018/05/08. 10.1194/jlr.C086512 29728460 PMC6027909

[2] Choi SH, Wallace AM, Schneider DA, Burg E, Kim J, Alekseeva E, et al. AIBP augments cholesterol efflux from alveolar macrophages to surfactant and reduces acute lung inflammation. JCI Insight. 2018;3(16). Epub 2018/08/24. 10.1172/jci.insight.120519 30135304 PMC6141166

[3] Dubrovsky L, Ward A, Choi SH, Pushkarsky T, Brichacek B, Vanpouille C, et al. Inhibition of HIV Replication by Apolipoprotein A-I Binding Protein Targeting the Lipid Rafts. mBio. 2020;11(1). Epub 2020/01/23. 10.1128/mBio.02956-19 31964734 PMC6974568

[4] Fang L, Choi SH, Baek JS, Liu C, Almazan F, Ulrich F, et al. Control of angiogenesis by AIBP-mediated cholesterol efflux. Nature. 2013;498(7452):118–22. 10.1038/nature12166 23719382 PMC3760669

[5] Fang L, Miller YI. Regulation of lipid rafts, angiogenesis and inflammation by AIBP. Curr Opin Lipidol. 2019;30(3):218–23. Epub 2019/04/16. 10.1097/MOL.0000000000000596 30985364 PMC6881232

[6] Gu Q, Yang X, Lv J, Zhang J, Xia B, Kim JD, et al. AIBP-mediated cholesterol efflux instructs hematopoietic stem and progenitor cell fate. Science. 2019;363(6431):1085–8. Epub 2019/02/02. 10.1126/science.aav1749 30705153 PMC6469354

[7] Low H, Mukhamedova N, Capettini L, Xia Y, Carmichael I, Cody SH, et al. Cholesterol Efflux-Independent Modification of Lipid Rafts by AIBP (Apolipoprotein A-I Binding Protein). Arterioscler Thromb Vasc Biol. 2020:ATVBAHA120315037. Epub 2020/08/14. 10.1161/ATVBAHA.120.315037 . 32787522 PMC7530101

[8] Mao R, Meng S, Gu Q, Araujo-Gutierrez R, Kumar S, Yan Q, et al. AIBP Limits Angiogenesis Through gamma-Secretase-Mediated Upregulation of Notch Signaling. Circ Res. 2017;120(11):1727–39. Epub 2017/03/23. 10.1161/CIRCRESAHA.116.309754 28325782 PMC5446274

[9] Schneider DA, Choi SH, Agatisa-Boyle C, Zhu L, Kim J, Pattison J, et al. AIBP protects against metabolic abnormalities and atherosclerosis. J Lipid Res. 2018;59(5):854–63. Epub 2018/03/22. 10.1194/jlr.M083618 29559522 PMC5928435

[10] Sorci-Thomas MG, Thomas MJ. AIBP, NAXE, and Angiogenesis: What’s in a Name? Circ Res. 2017;120(11):1690–1. Epub 2017/05/27. 10.1161/CIRCRESAHA.117.311023 28546345 PMC5497770

[11] Westerterp M. AIBP decreases atherogenesis by augmenting cholesterol efflux. Atherosclerosis. 2018;273:117–8. Epub 2018/04/28. 10.1016/j.atherosclerosis.2018.04.018 . 29699701

[12] Woller SA, Choi SH, An EJ, Low H, Schneider DA, Ramachandran R, et al. Inhibition of Neuroinflammation by AIBP: Spinal Effects upon Facilitated Pain States. Cell Rep. 2018;23(9):2667–77. Epub 2018/05/31. 10.1016/j.celrep.2018.04.110 . 29847797 PMC6239868

[13] Zhang M, Li L, Xie W, Wu JF, Yao F, Tan YL, et al. Apolipoprotein A-1 binding protein promotes macrophage cholesterol efflux by facilitating apolipoprotein A-1 binding to ABCA1 and preventing ABCA1 degradation. Atherosclerosis. 2016;248:149–59. Epub 2016/03/28. 10.1016/j.atherosclerosis.2016.03.008 . 27017521

[14] Zhang M, Zhao GJ, Yao F, Xia XD, Gong D, Zhao ZW, et al. AIBP reduces atherosclerosis by promoting reverse cholesterol transport and ameliorating inflammation in apoE(-/-) mice. Atherosclerosis. 2018;273:122–30. 10.1016/j.atherosclerosis.2018.03.010 PubMed PMID: WOS:000432773300019. 29555084

[15] Zhang M, Zhao GJ, Yin K, Xia XD, Gong D, Zhao ZW, et al. Apolipoprotein A-1 Binding Protein Inhibits Inflammatory Signaling Pathways by Binding to Apolipoprotein A-1 in THP-1 Macrophages. Circ J. 2018;82(5):1396–404. Epub 2018/04/06. 10.1253/circj.CJ-17-0877 . 29618705

[16] Zhang T, Wang Q, Wang Y, Wang J, Su Y, Wang F, et al. AIBP and APOA-I synergistically inhibit intestinal tumor growth and metastasis by promoting cholesterol efflux. J Transl Med. 2019;17(1):161. Epub 2019/05/19. 10.1186/s12967-019-1910-7 31101050 PMC6524272

[17] Zhu L, Parker M, Enemchukwu N, Shen M, Zhang G, Yan Q, et al. Combination of apolipoprotein-A-I/apolipoprotein-A-I binding protein and anti-VEGF treatment overcomes anti-VEGF resistance in choroidal neovascularization in mice. Commun Biol. 2020;3(1):386. Epub 2020/07/18. 10.1038/s42003-020-1113-z 32678293 PMC7367303

[18] Ritter M, Buechler C, Boettcher A, Barlage S, Schmitz-Madry A, Orso E, et al. Cloning and characterization of a novel apolipoprotein A-I binding protein, AI-BP, secreted by cells of the kidney proximal tubules in response to HDL or ApoA-I. Genomics. 2002;79(5):693–702. 10.1006/geno.2002.6761 . 11991719

[19] Becker-Kettern J, Paczia N, Conrotte JF, Zhu C, Fiehn O, Jung PP, et al. NAD(P)HX repair deficiency causes central metabolic perturbations in yeast and human cells. FEBS J. 2018;285(18):3376–401. Epub 2018/08/12. 10.1111/febs.14631 . 30098110

[20] Marbaix AY, Noel G, Detroux AM, Vertommen D, Van Schaftingen E, Linster CL. Extremely conserved ATP- or ADP-dependent enzymatic system for nicotinamide nucleotide repair. J Biol Chem. 2011;286(48):41246–52. 10.1074/jbc.C111.310847 21994945 PMC3308837

[21] Kremer LS, Danhauser K, Herebian D, Petkovic Ramadza D, Piekutowska-Abramczuk D, Seibt A, et al. NAXE Mutations Disrupt the Cellular NAD(P)HX Repair System and Cause a Lethal Neurometabolic Disorder of Early Childhood. Am J Hum Genet. 2016;99(4):894–902. Epub 2016/09/13. 10.1016/j.ajhg.2016.07.018 27616477 PMC5065653

[22] Trinh J, Imhoff S, Dulovic-Mahlow M, Kandaswamy KK, Tadic V, Schafer J, et al. Novel NAXE variants as a cause for neurometabolic disorder: implications for treatment. J Neurol. 2020;267(3):770–82. Epub 2019/11/21. 10.1007/s00415-019-09640-2 . 31745726

[23] Lee JS, Yoo T, Lee M, Lee Y, Jeon E, Kim SY, et al. Genetic heterogeneity in Leigh syndrome: Highlighting treatable and novel genetic causes. Clin Genet. 2020;97(4):586–94. Epub 2020/02/06. 10.1111/cge.13713 . 32020600

[24] Incecik F, Ceylaner S. Early-onset progressive encephalopathy associated with NAXE gene variants: a case report of a Turkish child. Acta Neurol Belg. 2020;120(3):733–5. Epub 2019/11/24. 10.1007/s13760-019-01242-z . 31758406

[25] Ruhoy IS, Saneto RP. The genetics of Leigh syndrome and its implications for clinical practice and risk management. Appl Clin Genet. 2014;7:221–34. Epub 2014/11/25. 10.2147/TACG.S46176 25419155 PMC4235479

[26] Choi SH, Agatisa-Boyle C, Gonen A, Kim A, Kim J, Alekseeva E, et al. Intracellular AIBP (Apolipoprotein A-I Binding Protein) Regulates Oxidized LDL (Low-Density Lipoprotein)-Induced Mitophagy in Macrophages. Arterioscler Thromb Vasc Biol. 2020:ATVBAHA120315485. Epub 2020/12/29. 10.1161/ATVBAHA.120.315485 PMID: 33356389 PMC8105271

[27] Niehaus TD, Elbadawi-Sidhu M, Huang L, Prunetti L, Gregory JF, 3rd, de Crecy-Lagard V, et al. Evidence that the metabolite repair enzyme NAD(P)HX epimerase has a moonlighting function. Biosci Rep. 2018;38(3). Epub 2018/04/15. 10.1042/BSR20180223 29654173 PMC5938422

[28] Marbaix AY, Tyteca D, Niehaus TD, Hanson AD, Linster CL, Van Schaftingen E. Occurrence and subcellular distribution of the NADPHX repair system in mammals. Biochem J. 2014;460(1):49–58. 10.1042/BJ20131482 . 24611804

[29] Ridlon JM, Kang DJ, Hylemon PB, Bajaj JS. Bile acids and the gut microbiome. Curr Opin Gastroenterol. 2014;30(3):332–8. Epub 2014/03/15. 10.1097/MOG.0000000000000057 24625896 PMC4215539

[30] Behrens WA, Thompson JN, Madere R. Distribution of alpha-tocopherol in human plasma lipoproteins. Am J Clin Nutr. 1982;35(4):691–6. Epub 1982/04/01. 10.1093/ajcn/35.4.691 . 7072621

[31] Goulinet S, Chapman MJ. Plasma LDL and HDL subspecies are heterogenous in particle content of tocopherols and oxygenated and hydrocarbon carotenoids. Relevance to oxidative resistance and atherogenesis. Arterioscler Thromb Vasc Biol. 1997;17(4):786–96. Epub 1997/04/01. 10.1161/01.atv.17.4.786 . 9108795

[32] Siems W, Muller M, Dumdey R, Holzhutter HG, Rathmann J, Rapoport SM. Quantification of pathways of glucose utilization and balance of energy metabolism of rabbit reticulocytes. Eur J Biochem. 1982;124(3):567–76. Epub 1982/06/01. 10.1111/j.1432-1033.1982.tb06631.x . 7106108

[33] Shajib MS, Baranov A, Khan WI. Diverse Effects of Gut-Derived Serotonin in Intestinal Inflammation. ACS Chem Neurosci. 2017;8(5):920–31. Epub 2017/03/16. 10.1021/acschemneuro.6b00414 . 28288510

[34] Yano JM, Yu K, Donaldson GP, Shastri GG, Ann P, Ma L, et al. Indigenous bacteria from the gut microbiota regulate host serotonin biosynthesis. Cell. 2015;161(2):264–76. Epub 2015/04/11. 10.1016/j.cell.2015.02.047 25860609 PMC4393509

[35] Shumilin IA, Cymborowski M, Chertihin O, Jha KN, Herr JC, Lesley SA, et al. Identification of unknown protein function using metabolite cocktail screening. Structure. 2012;20(10):1715–25. Epub 2012/09/04. 10.1016/j.str.2012.07.016 22940582 PMC3472112

[36] Choi SH, Kim KY, Perkins GA, Phan S, Edwards G, Xia Y, et al. AIBP protects retinal ganglion cells against neuroinflammation and mitochondrial dysfunction in glaucomatous neurodegeneration. Redox Biol. 2020;37:101703. Epub 2020/09/09. 10.1016/j.redox.2020.101703 32896719 PMC7484594

[37] Qiu X, Luo J, Fang L. AIBP, Angiogenesis, Hematopoiesis, and Atherogenesis. Curr Atheroscler Rep. 2020;23(1):1. Epub 2020/11/25. 10.1007/s11883-020-00899-9 . 33230630 PMC8941773

[38] Wrenger C, Eschbach ML, Muller IB, Warnecke D, Walter RD. Analysis of the vitamin B6 biosynthesis pathway in the human malaria parasite Plasmodium falciparum. J Biol Chem. 2005;280(7):5242–8. Epub 2004/12/14. 10.1074/jbc.M412475200 . 15590634

