# Supplementary material for: Systemic metabolite profiling reveals sexual dimorphism of AIBP control of metabolism in mice
Source: PLoS One. 2021 Apr 1;16(4):e0248964. doi: 10.1371/journal.pone.0248964 (PMC8016339; doi:10.1371/journal.pone.0248964)
Supplement: S1 File — (PDF) [file pone.0248964.s002.pdf]

S1 File

Systemic metabolite profiling reveals sexual dimorphism of AIBP control of metabolism in mice

Jun-dae Kim[a], Lingping Zhu[a,b], Quan Sun[a,b], and Longhou Fang[a,c,d, 1]

[a] Center for Cardiovascular Regeneration, Houston Methodist Research Institute,
[b]Department of Geriatric Medicine, Xiangya Hospital, Central South University, Changsha, Hunan, P.R. China.
[c]Department of Cardiovascular Sciences, 6550 Fannin St, TX77030, USA,
[d]Weill Cornell Medical College, 407 E 61st St, New York, NY 10065, USA

Running title: AIBP role in cellular metabolism

1: To whom correspondence should be addressed. Tel: 713-363-9012
Email: lhfang@houstonmethodist.org

S1 Fig

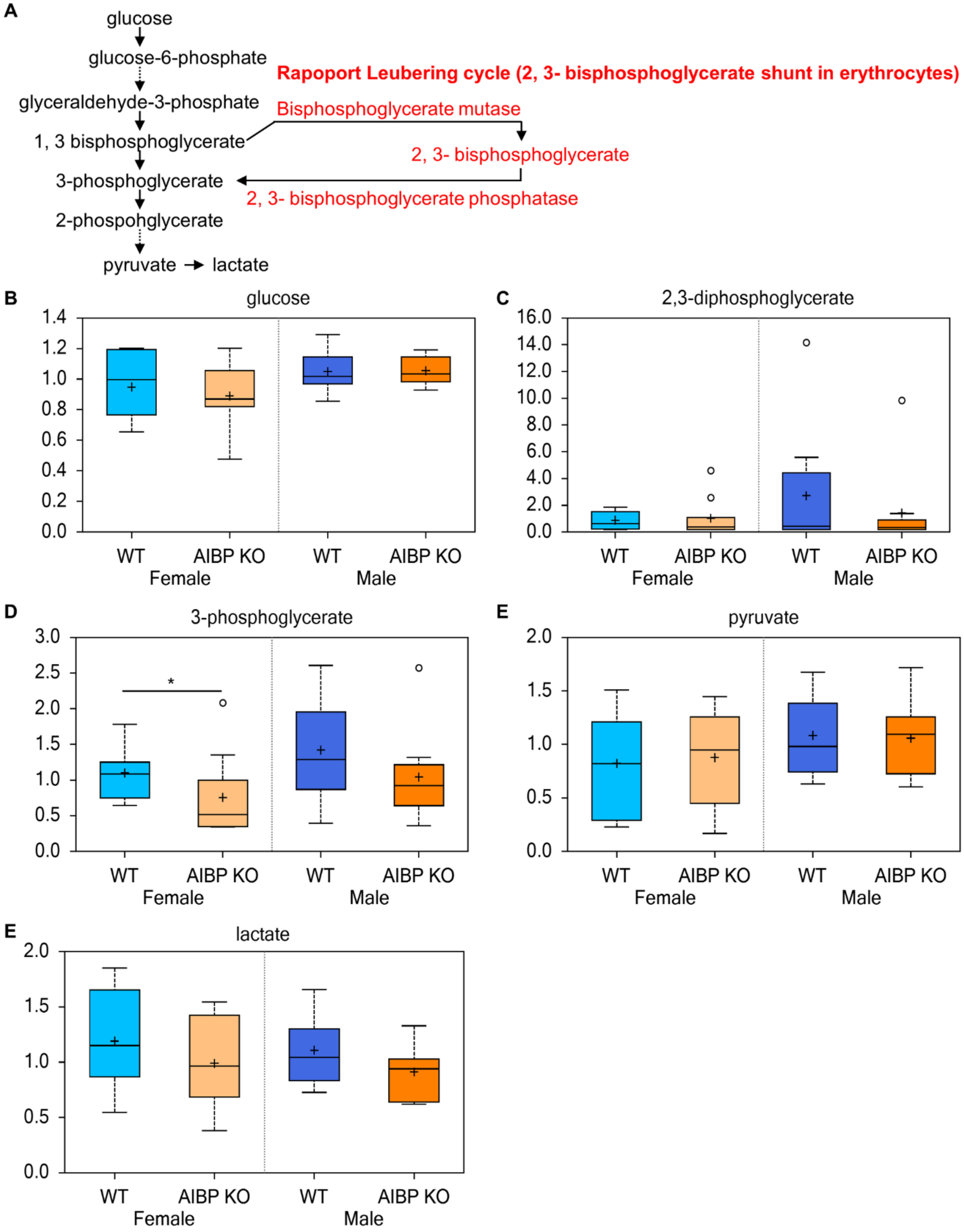
A
glucose
glucose-6-phosphate
glyceraldehyde-3-phosphate
1, 3 bisphosphoglycerate
3-phosphoglycerate
2-phospohglycerate
pyruvate
lactate
Rapoport Leubering cycle (2, 3- bisphosphoglycerate shunt in erythrocytes)
Bisphosphoglycerate mutase
2, 3- bisphosphoglycerate
2, 3- bisphosphoglycerate phosphatase
B
glucose
C
2,3-diphosphoglycerate
D
3-phosphoglycerate
E
pyruvate
E
lactate
WT
AIBP KO
Female
Male

19

S1 Fig. Loss of AIBP has no effect on glycolysis. **A**. Diagram illustration of glycolysis pathway. The particular shunt that occurs in red blood cells is shown in red. (**B-C**) Metabolites associated with glycolysis. Except 3-phosphoglycerate, all the rest metabolites are not significantly changed.

S2 Fig

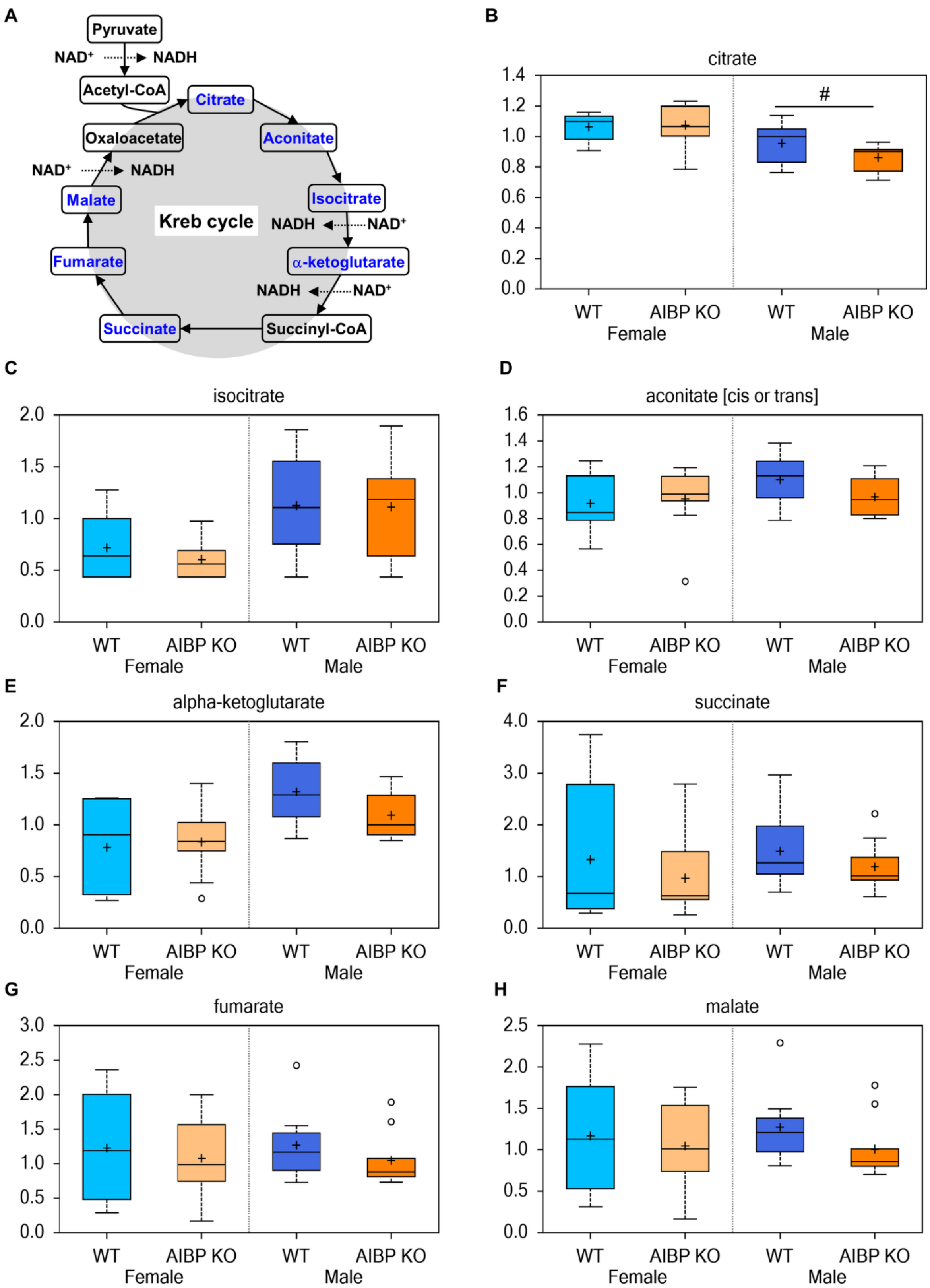

A
Pyruvate
NAD+
NADH
Acetyl-CoA
Citrate
Oxaloacetate
Aconitate
NAD+
NADH
Malate
Isocitrate
Kreb cycle
NADH
NAD+
Fumarate
α-ketoglutarate
NADH
NAD+
Succinate
Succinyl-CoA
B
citrate
#
C
isocitrate
D
aconitate [cis or trans]
E
alpha-ketoglutarate
F
succinate
G
fumarate
H
malate
WT
AIBP KO
WT
AIBP KO
Female
Male

24

S2 Fig. AIBP deficiency does not affect Krebs cycle. A. Diagram showing Krebs cycle progression. (**B-H**) Plasma metabolites associated with Krebs cycle. All changes did not reach statistically significant difference. $0.1>p>0.05$.
